# Misophonia in Children and Adolescents: Age Differences, Risk Factors, Psychiatric and Psychological Correlates. A Pilot Study with Mothers’ Involvement

**DOI:** 10.1007/s10578-023-01593-y

**Published:** 2023-09-08

**Authors:** Marta Siepsiak, Anna Turek, Magdalena Michałowska, Małgorzata Gambin, Wojciech Łukasz Dragan

**Affiliations:** 1 Faculty of Psychology, University of Warsaw, Warsaw, Poland; 2 Institute of Psychology, Jagiellonian University, Kraków, Poland

**Keywords:** Misophonia, Children, Adolescents, Psychopathology, Sensory sensitivity

## Abstract

Misophonia is a type of disorder characterized by decreased sound tolerance. While it typically begins in childhood, research on its characteristics in this population is limited. We assessed 90 children aged 7–18 with and without misophonia, along with their mothers, using interviews, questionnaires, and performance-based tests. Younger children with misophonia were more likely to use aggression in response to triggers than older, while adolescents largely reported self-harm during triggers. Children with misophonia did not differ from their peers in terms of ADHD, ODD, ASD, dyslexia, social and emotional competencies, head injuries, epilepsy, tinnitus, being prematurely born, or delivered via cesarean sections. However, they had significantly higher symptoms of anxiety and depression, more frequent occurrences of OCD, migraines, and psychosomatic complaints. Their mothers self-reported postpartum depression significantly more frequently than mothers in the control group. There is a need for further research on pediatric misophonia, with the involvement and assessment of parents.

**Supplementary Information:**

The online version contains supplementary material available at 10.1007/s10578-023-01593-y.

## Introduction

Misophonia is a disorder characterized by over-responsivity to certain repetitive stimuli, particularly sounds, which are often produced by humans, including chewing, breathing, humming, and sniffling [1]. These sounds, referred to as triggers, elicit feelings of irritation, anger, and sometimes disgust, anxiety, sadness, helplessness, or other emotions. Individuals with misophonia tend to avoid exposure to their triggers, and when avoidance is not possible, they experience intense distress [2, 3]. At the same time, there is currently no data to support the notion that habituation to these sounds is possible, as is the case in anxiety disorders. As a result, their quality of life can deteriorate and social functioning may become severely impaired. While misophonia has been observed to co-occur with various psychiatric disorders [3–8] or developmental disorders [9, 10] it cannot be attributed exclusively to any specific disorder.

Since misophonia usually develops during childhood or adolescence [11, 12], it is crucial to investigate the condition in this population to gain a more profound understanding of its development and progression and plan effective therapeutic and supportive interventions. However, there is currently limited knowledge on misophonia in youths, as almost all the research on this topic has been conducted on adults. Most of the knowledge on misophonia in children comes from several case studies, mostly reporting on patients referred to clinics [13–16]. The data indicates a great variability in the co-occurrence of psychiatric disorders in this group, and highlights the idiosyncratic nature of the problem, often discussed in terms of family and school. Nonetheless, it reveals a unique pattern of misophonia symptoms in children that is observable in adults as well.

In a recent study by [17, 18], 15 children with misophonia were identified from a sample of 142 children, and the findings showed that children with this disorder had a significantly lower quality of life compared to the overall population sample. Although the study had limitations, such as being questionnaire-based and conducted online, it provides a basis and reason for further exploration of this group. To date, only one study has investigated misophonia in a sample of children, and their parents using a combination of psychiatric face-to-face assessments and multiple questionnaires [6]. The findings of this study align with some previous research [7, 8] conducted on adult populations with misophonia, and reveals a high incidence of comorbid psychiatric disorders in children with misophonia. In Guzick et al.’s [6] study, 78% of children diagnosed with misophonia met the criteria for at least one psychiatric disorder, with depression and anxiety disorders being the most commonly observed. However, the findings regarding psychiatric comorbidities in misophonia are inconsistent across studies, especially regarding attention-deficit/hyperactivity disorder (ADHD). While some studies have reported higher rates, such as Guzick et al. [6] finding 21% of children with misophonia also had ADHD, or Kılıç et al. [19] reporting 20% of adult misophonia sufferers with ADHD, others have reported much lower rates. For example, only 5% of adult misophonia sufferers in the Netherlands were diagnosed with ADHD [3, 20, 21]. Conversely, Rosenthal et al. [7] found no relationship between ADHD and misophonia symptoms in an American sample. Due to the limited data and inconsistent findings on psychiatric comorbidities in misophonia, especially in children, our study aimed to investigate these factors in a sample of Polish-speaking children and teenagers.

Although 8–11% of the children in Guzick et al.'s [6] study exhibited clinically elevated symptoms of autism spectrum disorder (ASD), the average symptoms of ASD were not elevated in this group, and they were not found to be correlated with misophonia symptoms. In fact, the symptoms of ASD were significantly lower in children with misophonia compared to those with anxiety disorders. However, these findings are inconsistent with the data obtained by [17, 18], who reported higher symptoms of ASD in children with misophonia. Therefore, the relationship between misophonia and ASD in the pediatric population remains unclear and requires further investigation. To address the limitations, this study aimed to examine the understanding of social functioning and emotional regulation in children with misophonia. To achieve this, in this study not only we measured ASD symptoms using parent-reporting scales, but also, we used performance-based tests to assess the social and emotional competencies of both children with misophonia and control group without misophonia. To the best of our knowledge, no previous studies have used psychological performance-based tests (in contrast to questionnaires) to evaluate these factors in children with misophonia. Moreover, we used a series of questionnaires and conducted clinical face-to-face interviews to assess the presence of psychiatric disorders and the severity of anxiety and depressive disorders.

In addition to examining psychiatric and developmental comorbidities, which may offer insight into the underlying mechanisms of misophonia, it is crucial to also consider other factors such as pre- and postnatal conditions and maternal well-being [22–24] that could also play a role in development or maintenance of misophonia symptoms. Currently, there is a lack of information on such risk factors in misophonia. In this study, we assessed these aspects and compared its occurrence between individuals with misophonia and controls without any sound over-responsivities, using mother-reported data, including gathering data on the occurrence of postpartum depression following the birth of the assessed child.

For a better understanding of the mechanisms of misophonia, it is also crucial to establish a comprehensive description of the clinical presentation of this disorder in its early stages, including its progression and changes throughout childhood and adolescence. This information is vital for early detection and intervention, potentially leading to improved outcomes for those affected by misophonia. No studies examining age-related misophonia characteristics have been published so far. Therefore, in this study, we also compared the clinical presentation of misophonia in younger children and teenagers, including the presence of aggressive behavior and self-harm in response to trigger sounds, as well as coping strategies.

In summary, the main aim of this pilot study was to evaluate a broad range of preliminary findings from previous research on misophonia within a Polish sample of children and adolescents and develop new hypotheses to test in further studies. Specifically, the main objectives of this study were to:

- describe the characteristics of misophonia in children and adolescents, including the age of onset, types of triggers, coping strategies, typical reactions to trigger sounds, emotional experiences, and reported direction of symptom development over time,
- investigate differences in aggressive and self-harming behaviors and coping skills between younger children (aged 7–12) and teenagers (aged 13–18) with misophonia,
- examine differences in perinatal characteristics, as well as somatic and psychosomatic complaints, between children with and without misophonia,
- investigate differences in the severity of depressive and anxiety symptoms, as well as in the occurrence of ASD, ADHD, ODD, OCD, and tic disorders, between children with and without misophonia,
- verify the emotional and social competencies of the assessed children with the performance-based tests,
- investigate whether there were any differences in the occurrence of stressful events during pregnancy and post-partum depression between mothers of children with and without misophonia,
- describe the prevalence of misophonia and autism in other family members of children with misophonia.

## Methodology

### Participants

A total of 90 children and teenagers between the ages of 7 and 18 (*M* = 12.6; *SD* = 3) participated in this study, along with their mothers. In some cases, fathers also participated in the interviews, and in one instance, a father was interviewed. All participants spoke Polish. The children were either attending school or being homeschooled and lived in Poland with at least one parent. The exclusion criteria for both the misophonia and control groups were: a diagnosis of autism spectrum disorder (ASD; official diagnosis reported by parents), intellectual disability (parent's report of official diagnosis and/or IQ screening test that was performed during the study; the data are not discussed in this paper as they are part of another manuscript), serious somatic illness (reported by parents at the time of phone screening), hearing loss (assessed using a screening audiometer; all children scored within the normal range of pure tone air conduction thresholds at all 8 frequencies, from 250 Hz to 8 kHz), or serious sight impairment (reported by parents at the time of phone screening). Because various forms of decreased sound tolerance, inlcuding misophonia, are very common in ASD [25], and social and emotional difficulties are among the main characteristics of ASD, while one of the main goals of this study was to assess these aspects, it was decided to exclude children with ASD to avoid potential biases.

Seven children were excluded from the analysis due to not meeting the study criteria or a significant amount of missing data. In the result, the misophonia group consisted of 45 children and teenagers, while the control group consisted of 38 participants without sound sensitivity issues. There was no significant age difference between the misophonia group (*n* = 44, *M* = 13.1, *SD* = 3) and the control group (*n* = 37, *M* = 11.9, *SD* = 2.9), *p* = 0.067; Cohen’s *d* = 0.411. There was also no significant gender difference between the misophonia group (68.2% female) and the control group (51.4% female), *p* = 0.123, χ^2^ = 2.383, *OR* = 2.030 [0.822, 5.015].

### Procedure

The study was approved by the Ethics Committee of the Faculty of Psychology at the University of Warsaw, with the Reference Number 10/06/2022. It was conducted between June 2022 and September 2022. To recruit participants, the study was advertised on social media, through radio broadcasts, and via email to parents who had previously expressed an interest in being informed about research studies. Interested parents were directed to an online form, which asked them to provide information about their child's over-responsivity to certain sounds, or if their child had no sound sensitivity issues, to indicate it too. They were subsequently contacted by phone for a short screening to assess their eligibility for the study. The Jager et al. [3] criteria for misophonia were used as a guideline. For instance, if a parent reported that their child was over-responsive to only loud sounds or sounds such as sirens, vacuum cleaners, or toys, the child was not invited to participate. As children may not be able to name the emotions they experience or their parents may not know what type of emotion their child could feel in response to the triggers, the emotional response type was not taken into consideration during the screening process. However, this was included as part of the main study for descriptive statistics (but not for misophonia diagnosis) when participants were met in person.

The study was conducted either at the Faculty of Psychology at the University of Warsaw (Poland) or at participants' homes located in 10 Polish voivodeships across all Poland, ranging from villages with as few as less than 199 inhabitants to the main Polish cities with populations of up to 1.765 million. In a few cases, the study was conducted online, and in such cases, it was limited to interview and questionnaire assessments (no performance-based tests).

The missing data, such as symptoms of depression and anxiety or emotional and social competencies tests, were due to factors such as a child being too tired to complete the assessments, a child being unable to fill a questionnaire or refusing it, or sometimes also time constraints. The average duration of the whole assessment was around 2 h.

In addition to the assessments described below, the children also underwent cognitive performance-based assessment, hearing tests, and central auditory processing tests. However, the analyses of these data are beyond the scope of the current study.

### Assessment

#### Misophonia Assessment

The misophonia assessment in this study was conducted by a psychologist familar with misophonia through face-to-face interviews, which were mainly based on the criteria established by [3]. For younger children, the mothers were the main source of the information about misophonia, but in all cases where the child was old enough and expressed interest, the child was also involved in the misophonia interview process. In the case of teenagers, they were the primary informants regarding their own misophonia, complemented by additional insights obtained through consultation with their mothers. This approach allowed for a more accurate and comprehensive assessment of the child's symptoms, including their onset.

The aim of this study was to identify children whose main trigger for misophonia was human oral or nasal sounds that elicited strong, unpleasant emotional reactions. However, we did not exclude any children based on their failure to report anger or irritation as the primary emotion. This was due to the possible variability in emotional expression [1] and the potential difficulties in accurately identifying and labeling emotions in children. In addition, significant impairment of functioning during exposure to the triggers was ascertained through reports of active avoidance or complaints, such as asking to stop making sounds, crying, leaving the room, or various types of aggression in response to the trigger sounds.

#### Pre, Perinatal Factors, and Other Medical and Health History Reports

The mothers provided oral information on their child's perinatal history, stress during pregnancy with the assessed child, birth complications, occurrence of postpartum depression, as well as information about the child's health complaints, such as migraines/strong headaches, psychosomatic symptoms (somatic complaints without documented medical reasons, such as unexplained stomach pain, back pain, or pain in other parts of the body, were considered; the physiological component of emotions, such as stomach pain right before or during an exam, was not counted as psychosomatic), head injuries, epilepsy, and dyslexia. Additionally, we asked about the presence of autism in the family, including the child's siblings, parents, grandparents, and uncles/aunts (i.e., the parent's siblings who shared at least one grandparent with the assessed child).

The data was recorded in a form using nominal scales, indicating the occurrence or absence of each event. The translation of the interview, created by the first author of the study, can be found in Supplementary File 1.

#### Age Group Division for Comparing Misophonia Characteristics

Due to the potentially debatable nature of how the age group should be created, we decided to divide the group based on the normative data presentation for depression symptoms in The Children’s Depression Inventory used in this study [26]. Specifically, the younger group included children up to 12 years old, while the older group comprised teenagers aged 13–18.

#### Symptoms of Anxiety

The parent version of the Spence Children's Anxiety Scale (SCAS; [27]) was used for all participants. However, in cases where the child was able to read and expressed an interest in participating, they completed a parallel version of this tool—the self-reported version of the questionnaire for children. As a result, there were two versions of the SCAS used in this study: the parent's version and the child's version. The SCAS is a widely used measure for assessing anxiety symptoms in children. It is a 38-item questionnaire with a four-point scale to assess the frequency of certain symptoms or situations. It consists of six scales that assess different types of anxiety: panic and agoraphobia, separation anxiety, fear of physical injuries, social phobia, obsessive–compulsive disorder (OCD), and generalized anxiety disorder (GAD), as well as a total outcome score. In a group of Polish teenagers as self-reported [28] Cronbach’s alphas for the separate scales ranged between 0.91 (for panic disorder and agoraphobia symptoms) and 0.77 (for social phobia symptoms).

#### Symptoms of Depression

The Children’s Depression Inventory (CDI 2; [29], polish adaptation: [30]) was used for identifying cognitive, affective and behavioral signs of depression in participants (between the ages of 7 and 18). In this study, we utilized the self-report short version for children, which consisted of one scale, and the 17-item parent-report version, which encompassed two scales. Satisfactory psychometric properties are reported for both versions: self-report (*α* = 0.74 in the group of Polish children aged 7–12, and *α* = 0.80 in the group of Polish adolescents aged 13–18) and parent-report (*α* = 0.84 for all of the ages, [30]).

#### Emotional and Social Competencies

Emotional and social competencies were assessed using the Intelligence and Development Scales for Children and Adolescents (IDS-2; [31, 32]). It is a performance-based test where children are asked: (a) to name the emotions shown in the pictures, (b) how they would regulate their emotions in various situations, and (c) how one should react in various social situations. The scoring was done according to the instructions provided by the authors of the tests. The reliability of this performance-based general socio-emotional competence index is satisfactory, with a score of about 0.80 in most groups [32].

#### Attention Deficit Hyperactivity Disorder and Oppositional Defiant Disorder

The structured diagnostic interview questionnaire developed by Wolańczyk and Kołakowski [33] was conducted with the mothers to assess attention deficit hyperactivity disorder (ADHD) and oppositional defiant disorder (ODD) in the children (polish version of the interview is attached in Online Appendix 2). The interview is commonly used in Poland (E.g. [34]. It is based on the ICD-10 criteria and comprises 18 items, with the severity of symptoms evaluated using a 4-point Likert scale. A score of six points (out of nine) on the Attention Deficit scale and at least six points (of nine) Hyperactivity-Impulsiveness (at least 3 out of 5 on the Hyperactivity scale and 1 out of 4 on the Impulsiveness sclae) scale is considered sufficient for an initial diagnosis of ADHD. For ODD, a minimum of four (out of eight) symptoms must be present for a diagnosis to be considered. In order to compare the data with previous misophonia studies that used the DSM-5, additionally we presented the rescored data for DSM-5 criteria.

#### Symptoms of Autism Spectrum Disorders

The Autism Spectrum Rating Scales (ASRS) is an autism screening tool developed by Goldstein and Naglieri [35]. In this study, the parent-rated version for children between 6 and 18 years old was utilized. The ASRS is designed to identify symptoms, behaviors, and associated features of ASD. Each item is rated on a 5-point Likert scale, with a higher total score indicating a greater intensity of ASD-type behaviors. The reliability of the ASRS main scales is reported to be high, with an alpha coefficient of over 0.8 for the parent version [30].

#### Obsessive–Compulsive Disorder and Tic Disorder Assessment

To assess the occurrence of OCD, a list of symptoms of obsessions and compulsions was taken from The Children’s Yale-Brown Obsessive–Compulsive Scale [36, 37]. Before asking a parent about the occurrence of the symptoms from the list, the definitions from the CY-BOCS of "obsessions" and "compulsions" were read to them. The clinician then ensured that the parent understood the definition and answered any questions if needed. Each time the parent named a symptom, the psychologist asked for examples to confirm the clinical meaning of the symptoms. OCD was reported as occurring in the case of the presence of obsessions, compulsions, or both for at least two weeks, when significant distress or interference with daily activities (ICD-10) was reported by the parent and supported with relevant examples. The scale for assessing the severity of the symptoms was not performed due to time constraints.

Tic disorder was assessed using the list of tics from the Yale Global Tic Severity Scale (YGTSS; [38], Polish adaptation: [39]), according to the ICD-10 criteria. This meant that tics were considered present if they persisted for at least 4 weeks, occurring on most days, and multiple times per day. The presence of tics was assessed solely based on parent reports, without direct observations. Due to time constraints, the questionnaire for assessing the severity of tic symptoms was not used.

## Results

### Statistical Analysis

The data were checked for normality, and in all cases, the skewness and kurtosis were between ± 1, and therefore parametric tests were used for analysis.

Regarding the characteristics of misophonia, descriptive statistics were run to evaluate frequencies of certain symptoms and behaviors for the entire misophonia group (I), and additionally, a Pearson bivariate correlation analysis was conducted to examine the reported age and the age of the assessed child. To compare misophonia symptoms across age groups (II), Chi-square Pearson tests with two-sided asymptotic significance were used to compare younger and older children with misophonia. To compare the occurrence of perinatal events and reported medical issues, as well as occurrence of psychiatric disorders (IIIa, b, c, f), Chi-square Pearson tests with two-sided asymptotic significance were conducted between children with and without misophonia. For analyzing the data on performance-based tests of emotional regulation and social functioning, as well as differences in the severity of symptoms of psychopathology (IIIb, d, e), t-tests with Welsh correction for independent samples were conducted.

#### Characteristic of Misophonia in the Entire Misophonia Group

The onset of misophonia symptoms before the age of 7 was reported by 50% of the parents of the children. Four parents reported that the first symptoms of misophonia were present already at the age of 3. For all of these 4 cases, the first triggers were eating sounds. A positive correlation between the age of the assessed child and the reported age of onset of misophonia symptoms was found: r = 0.525, *p* < 0.001.

Oral human-made sounds were a primary trigger for all the participants because it was the main inclusion criteria. Additionally, 63% were triggered by sniffling sounds, 59% by breathing sounds, and 27% by loud sounds, 18% sudden sounds, 46% sounds behind the wall, and 21% sounds of normal chatting. Among the other named sounds, the most frequent were: snoring (18%), whispering (16%), singing (16%), repetitive tapping (14%), clutter clink (11%), typing (7%), radio (7%), humming (6%).

The most common emotion in response to trigger was anger (89%), which was followed by irritation (84%), disgust (57%), frustration (57%), concern (33%), panic (30%), anxiety (16%), and fear (2%)—multiple answers were allowed.

According to the parents, 64% of the children’s first trigger was made by a close family member, and for 11%—other persons. For 50% of the children, the family member was currently the main trigger. For 57% of children misophonic reactions get worse over time, and for 46% the number of the triggers increased over time.

54% of parents of the assessed children with misophonia reported a family member having misophonia symptoms (35% parents, 7% siblings, 23% grandparents, 23% uncles/aunts).

#### Age Differences in Misophonia Clinical Picture—Comparison of Younger (7–12) and Older (13–18) Children and Teenagers with Misophonia

Younger children with misophonia were significantly more likely than teenagers to use verbal and physical aggression in response to trigger sounds than teenagers. Sixty-five percent of younger children (in comparison to 26% in the teenagers group) were reported by their parents to frequently shout at a person making trigger sounds χ^2^ (1, *N* = 44) = 6.490, *p* = 0.011; *OR* = 5.238 [1.406, 19.519]), and 41% of younger children, in comparison to 7% of the older ones, were reported to use physical aggression such as kicking, punching, or pushing a person making the trigger sounds (χ^2^ (1, *N* = 44) = 7.311, *p* = 0.007); *OR* = 8.750 [1.545, 49.562].

Instead, teenagers were more likely than the younger children to harm themselves during the expositions to the trigger sounds (χ^2^ (1, *N* = 44) = 8.590, *p* = 0.003; *OR* = 0.067 [0.008, 0.582]; 48% vs. 6%). Pinching, scratching skin, pulling out hairs, biting lips, among others were reported. Bruises, scratches, and bleeding were also reported as a result of these actions.

There were no age differences in the presence of emotional reaction before the triggers are present—the anticipation was reported by 70% of participants in both groups. The age groups did not differ in terms of coping strategies such as headphones, leaving the room or covering ears in the presence of the trigger sounds. The detailed data on the differences between younger and older children are presented in Table 1.

**Table 1:** Difference between younger and older children with misophonia

Misophonia			
	Younger children: 7–12 year old *N*(%)	Teenagers: 13–18 year old *N*(%)	χ^2^ (younger children vs. teenagers)
Anticipation emotional reaction	12 (70.6)	19 (70.4)	χ^2^(2, *N* = 44) = .000, *p* = .988; *OR* = 1.011 [.267, 3.824]
Asking politely to stop making the sound	5 (29.4)	14 (51.9)	χ^2^(1, *N* = 43) = 2.141, *p* = .143; *OR* = .387 [.107, 1.402]
Shouting to stop making the sound	11 (64.7)	7 (25.9)	χ^2^(1, *N* = 44) = 6.490, ***p* = .011;** ***OR* = 5.238 [1.406, 19.519]**
Leaving the place because of the sound	12 (70.6)	23 (85.2)	χ^2^ (1, *N* = 44) = 1.366, *p* = .242; *OR* = .417 [.094, 1.849]
Aggression toward objects in response to the sound exposition	10 (58.8)	6 (22.2)	χ^2^ (1, *N* = 44) = 6.039, ***p* = 0.014; *OR* = 5.00 [1.329, 18.814]**
Physical aggression toward other people in response to the sound exposition	7 (41.2)	2 (7.4)	χ^2^ (1, *N* = 44) = 7.311, ***p* = .007; *OR* = 8.750 [1.545, 49.562]**
Self-harm in response to the sound exposition	1 (5.9)	13 (48.1)	χ^2^ (1, *N* = 44) = 8.590, ***p* = .003;** *OR* = .067 [.008, .582]
Ear covering	11 (64.7)	15 (55.6)	χ^2^ (1, *N* = 44) = 0.361, *p* = .548; *OR* = 1.467 [.420, 5.126]
Headphones	9 (52.9)	21 (77.8)	χ^2^ (1, *N* = 44) = 2.966, *p* = .085; *OR* = .321 [.086, 1.198]

Statistically significant differences are bolded

#### Differences Between the Characteristics of Children With and Without Misophonia, and Their Mothers

##### Maternal Postpartum Depression, Pre and postnatal Children Characteristics

Significantly more mothers of children with misophonia χ^2^ (1, *N* = 81) = 5.853; *p* = 0.016; *OR* = 9.257 [1.114, 76. 947]; 20.5% vs. 2.7%, reported occurrence of postpartum depression. There were no differences in the mothers between the two groups in reported perinatal factors, such as labor, birth complications, reported increased stress during pregnancy nor prematurity of the child (for details see Table 2).

**Table 2:** Difference in clinical characteristics between children with and without misophonia and their mothers

	Misophonia *n* (%)^a^	Controls *n* (%)	χ^2^ (Misophonia vs. Control)
Female	30 (68.2)	19 (51.4)	χ^2^ (1, *N* = 81) = 2.38, *p* = .123, *OR* = 2.030 [.822, 5.015]
Postpartum depression in mother, pre and perinatal characteristics			
Mother’s postpartum depression	9 (20.5)	1 (2.7)	χ^2^ (1, *N* = 81) = 5.853, ***p* = .016; *OR* = 9.257 [1.114, 76. 947]**
Birth: C—section	11 (25)	5 (13.5)	χ^2^ (1, *N* = 81) = 1.67, *p* = .196; *OR* = 2.113 [.666, 6.830]
Birth: premature	4 (9.1)	2 (5.4)	χ^2^ (1, *N* = 81) = 0.398, *p* = .528; *OR* = 1.750 [.302, 10.141]
Birth complications	12 (27.9)	6 (16.2)	χ^2^ (1, *N* = 80) = 1.559, *p* = .212; *OR* = 2.0 [.666, 6.003]
Stress during pregnancy (mother)	10 (22.7)	6 (16.2)	χ^2^ (1, *N* = 81) = 0.538, *p* = .463; *OR* = 1.520 [.494, 4.672]
Neurological, developmental, and (neuro)psychiatric characteristics			
Migraines (child)^b^	12 (27.3)	2 (5.4)	χ^2^ (1, *N* = 81) = 6.722, ***p* = .010; *OR* = 6.563 [1.363, 31.601]**
Psychosomatic complaints^b^	14 (34.1)	3 (8.3)	χ^2^ (1, *N* = 77) = 7.424, ***p* = .006; *OR* = 5.704 [1.484, 21.929]**
OCD^b^	6 (13.6)	0	χ^2^ (1, *N* = 81) = 5.449, ***p* = .020**
Increased self-reported depressive symptoms^c^	18 (60)	7 (25)	χ^2^ (1, *N* = 58) = 7.234, ***p* = .007; *OR* = 4.50 [1.461, 13.859]**
Increased parent -reported overall depressive symptoms^c^	24 (58.5)	11 (34.4)	χ^2^ (1, *N* = 73) = 4.204, ***p* = .040; *OR* = 2.695 [1.034, 7.026]**
Tic disorder^b^	8 (18.2)	2 (5.4)	χ^2^ (1, *N* = 81) = 3.032, *p* = .082; *OR* = 3.889 [.771, 19.608]
Tinnitus (child)^b^	1 (2.3)	0	χ^2^ (1, *N* = 81) = 0.851, *p* = .356
Autism in family	13 (29.5)	6 (16.2)	χ^2^ (1, N = 81) = 1.989, *p* = .158; *OR* = 2.167 [.730, 6.431]
ADHD (ICD-10)	2 (4.8)	1 (2.7)	χ^2^ (1, *N* = 79) = 0.228, *p* = .633; *OR* = 1.80 [.157, 20.699]
ADHD any type (DSM-5)	11 (26.2%)	6 (16.2%)	χ^2^ (1, *N* = 79) = 1.159, *p* = .282; *OR* = 1.883 [.603, 5.576]
ADHD combined	3 (7.1%)	1 (2.7%)	χ^2^ (1, *N* = 79) = ,807, *p* = .369; *OR* = 2.769 [.275, 27.844]
ADHD inattentive	7 (16.7%)	4 (10.8%)	χ^2^ (1, *N* = 79) = 1.508, *p* = .471; *OR* = 1.650 [.442, 6.160]
ADHD hyperactive/impulsive	1 (2.4%)	1 (3%)	χ^2^ (1, *N* = 79) = .810, *p* = .667; *OR* = .878 [.053, 14.551]
ODD (ICD-10)	0	0	−
Dyslexia	5 (11.4)	3 (8.1)	χ^2^ (2, *N* = 81) = 2.605, *p* = .272; *OR* = 1.386 [.303, 6.164]
Epilepsy (child)	2 (4.5)	0	χ^2^ (1, N = 81) = 1.724, *p* = 0.189
Head Injuries^d^	2 (4.5)	3 (8.1)	χ^2^ (1, *N* = 81) = 0.440, *p* = .507; *OR* = .540 [.085, 3.417]

Statistically significant differences are bolded

^a^Percent of positive responses within a group, ^b^lifetime or current, ^c^60 or higher on CDI-2 ten scale, ^d^with loss of consciousness, vomiting or dizziness; in misophonia group—before misophonia symptoms onset, in controls any time

##### Developmental and Conduct Disorders, Dyslexia, Emotional and Social Competencies

There were no differences between the groups in the number of children who met the diagnostic criteria for ADHD and ODD, regardless of the chosen criteria (ICD-10 or DSM-5), and who had a report of dyslexia.

Statistical analysis revealed no significant differences in ASD symptoms between the two groups (for detailed statistics, see Table 3). Additionally, there were no significant differences in the reported frequency of autism diagnosis in families. It is worth noting however, that the occurrence of autism in families was high in both groups, with 29.5% in the misophonia group and 16.2% in the control group.

**Table 3:** Group differences in Symptoms of Autism Spectrum Disorder and Social and Emotional Competencies

Symptoms of autism spectrum disorder ASRS	Misophonia (*n* = 43)		Control (*n* = 34)		*t*	*df*	*p*	Cohen’s* d*
	*M*	*SD*	*M*	*SD*				
Social/communication	50.91	12.08	49.79	11.62	0.410	72.12	0.683	0.094
Unusual behaviors	50.84	12.06	48.59	10.56	0.871	74.17	0.386	0.197
Self-regulation	54.88	10.08	50.35	10.96	1.87	67.98	0.066	0.433
Peer socialization	52.48	10.33	53.38	10.49	− 0.374	70.44	0.710	− 0.086
Adult socialization	52.28	10.60	50.97	9.24	0.58	74.25	0.565	0.131
Social/emotional reciprocity	52.81	10.69	50.47	11.00	0.940	70.00	0.351	0.216
Atypical language	48.00	8.90	47.74	9.73	0.123	67.80	0.902	0.029
Stereotypy	46.42	8.85	46.26	10.20	0.070	65.68	0.945	0.016
Behavioral rigidity	49.77	10.56	48.59	10.20	0.496	72.00	0.623	0.113
Sensory sensitivity	56.93	10.96	52.32	9.89	1.94	73.66	0.057	0.439
Attention	54.42	11.26	51.62	12.87	1.00	66.00	0.320	0.234

Social and emotional competencies IDS-2	(*n* = 38)		(*n* = 32)					
Emotional regulation	11.00	2.79	11.19	2.83	− 0.278	65.63	0.782	− 0.067
Social strategies	10.39	3.47	11.50	4.13	− 1.20	60.81	0.235	− 0.292

Furthermore, performance-based IDS-2 tasks measuring emotional and social competencies did not reveal any significant differences between children with and without misophonia (for statistical analysis, see Table 3).

##### Migraine, Head Injuries, Epilepsy, Tinnitus, and Psychosomatic Complaints

Children with misophonia significantly more often than controls were reported to experience migraines or strong headaches (27.3% vs. 5.4%; χ^2^ (1, *N* = 81) = 6.722; *p* = 0.010; *OR* = 6.563 [1.363, 31.601]), and psychosomatic complaints (34.1% vs. 8.3%; χ^2^ (1, *N* = 77) = 7.424; *p* = 0.006; *OR* = 5.704 [1.484, 21.929]). No group differences were found in reported epilepsy, head injuries and tinnitus. For details see Table 2.

##### Symptoms of Depression

When comparing the depressive symptoms between groups on all the 4 scales (3 reported by parents and one self-report; CDI-2), children with misophonia (*n* = 30, *M* = 62; *SD* = 9.33) had higher depressive symptoms than controls (*n* = 28, *M* = 54.21; *SD* = 9.97) only in self-report *t*(56) = 3.099; *p* = 0.003; Cohen’s *d* = 0.814 (see the detailed data in Table 4), while there were no differences in mothers’ reports of the child symptoms. Nonetheless, significantly more children in misophonia group had clinically elevated depressive symptoms (scored 60 or higher in ten scale of CDI-2) not only in the self-report assessment (χ^2^ (1, *N* = 58) = 7.234; *p* = 0.007; *OR* = 4.50 [1.461, 13.859]; 60% vs. 25%), but also in the parent report assessment (χ^2^ (1, *N* = 73) = 4.204; *p* = 0.040; *OR* = 2.695 [1.034, 7.026]; 58.5% vs. 34.4%).

**Table 4:** Group differences in depression and anxiety symptoms

Symptoms of anxiety disordres SCAS parent-rated	Misophonia (n = 42)		Controls (n = 35)		*t*^a^	*df*	*p*	Cohen’s *d*
	*M*	*SD*	*M*	*SD*				
Panic and agoraphobia	2.16	2.94	1.11	1.66	1.97^a^	66.61	0.052	0.431
Separation anxiety	3.50	2.89	3.03	2.41	0.78	75.00	0.44	0.176
Fear of physical injouries	3.48	2.75	2.94	2.17	0.95	74.79	0.345	0.213
Social phobia	6.95	3.56	5.63	3.13	1.74	74.78	0.087	0.393
OCD	1.98	2.27	1.20	1.30	1.88	64.16	0.065	0.410
GAD	5.05	2.99	3.57	2.34	2.43	74.75	**.018**	**.544**

Symptoms of anxiety disordres SCAS child-rated	(n = 33)		(n = 25)					
Separation anxiety	4.47	2.53	3.20	2.69	1.83	50.06	0.074	0.488
Social phobia	8.40	3.44	6.56	3.15	2.11	53.92	**0.039**	**0.553**
OCD	6.12	3.61	4.80	3.43	1.42	53.15	0.161	0.374
Panic and agoraphobia	6.21	5.15	3.44	3.56	2.42	55.61	**0.019**	**0.611**
Fear of physical injouries	4.58	2.14	3.60	2.22	1.69	50.79	0.098	0.449
GAD	8.58	3.36	5.88	2.91	3.27	54.97	**0.002**	**0.849**

Symptoms of depression parent-rated CDI-2	(n = 41)		(n = 32)					
Total score	59.07	12.11	55.81	10.76	1.22	69.76	0.228	0.282
Emotional problems	58.41	13.24	54.75	10.88	1.30	70.79	0.199	0.299
Functional problems	56.80	11.72	56.06	10.13	0.290	70.21	0.773	0.067

Symptoms of depression child-rated CDI-2	(n = 30)		(n = 28)					
Total score	62.07	9.33	54.21	9.97	3.09	54.97	**0.003**	**0.814**

Statistically significant differences are bolded

^a^For all the tests, equal variances were not assumed

##### Symptoms of Anxiety Disorders

A total of 12 scales (i.e. 6 self-reported symptoms and the same 6 symptoms reported by parents) were analyzed separately: separation anxiety, social phobia, panic and agoraphobia (one scale), fear of physical injuries, OCD and GAD. Children with misophonia had higher severity of symptoms of GAD in both—parent-report *t*(75) = 2.376; *p* = 0.020; Cohen’s *d* = 0.544, and self-report *t*(56) = 3.202; *p* = 0.002; Cohen’s *d* = 0.849. Additionally, children with misophonia had higher self-reported symptoms of panic and agoraphobia *t*(56) = 2.422; *p* = 0.019; Cohen’s *d* = 0.611 and social phobia *t*(56) = 2.085, *p* = 0.042; Cohen’s *d* = 0.553. For the detailed statistics see Table 4.

##### Obsessive–Compulsive Disorder and Tic Disorder

Significantly more children with misophonia met the criteria for OCD.

χ^2^ (1, *N* = 81) = 5.449; *p* = 0.020; 13.6% vs. 0%. While tics disorders appeared in misophonia almost 4 times more frequently than in controls (18.2% vs. 5.4%), there was no statistically significant difference between the groups. For the detailed statistics see Table 2.

## Discussion

This is one of the first studies on the characteristics of misophonia in children, and the first one to examine differences in misophonia characteristics between a group of younger individuals (aged 7–12) and older ones (aged 13–18). Additionally, it is novel here to investigate pre- and perinatal characteristics of such children, along with the occurrence of postpartum depression in their mothers. The entire misophonia sample was also compared to their non-misophonia peers in terms of health characteristics and emotional and social skills.

In the misophonia group, according to retrospective reports from the mothers, half of our sample exhibited the full symptoms of misophonia by the age of 7, with four children possibly meeting the criteria as early as at the age of 3 years. This suggests that misophonia may begin even earlier than previously reported [5, 12]. If misophonia is observed in infants or toddlers, new ways of assessment appropriate for these age groups should be carefully developed. It may be more difficult to diagnose and distinguish misophonia from hyperacusis or other forms of decreased sound tolerance in children who are still not fully able to verbalize their needs than it is in school-aged youth. Furthermore, the correlation between the age of onset and the age of the assessed child suggests that individuals assessed at a later age may be less likely to recall early experiences with misophonia. These findings emphasize the significance of studying children with misophonia and involving their parents. Early detection of misophonia and well-planned and executed interventions may potentially prevent or mitigate the adverse long-term effects of misophonia on mental health and family life, which warrants careful investigation in future studies.

Although the first trigger for the majority of children (64%) was a sound made by a family member, for the rest of the group, it was either a non-family member or both (family and non-family). Moreover, when asked about their current main or worst triggers, this percentage dropped, and only 50% of children were mainly triggered by family members. These results confirm notions that sounds made by family members are discerned triggers, but also show that it is not an indispensable characteristic of misophonia. The high percentage of participants for whom the first trigger was a family member may be explained by the fact that children spend most of their time with family members, and specific, repetitive sounds triggering misophonia are more likely to occur in proximity.

In this study, we explored for the first time the differences in clinical presentation of misophonia in younger and older age groups of children and adolescence. Our findings revealed that physical and verbal aggression is very common in misophonia, but only in younger children aged 7–12 years old. Physical aggression was reported in only 7% of the teenage group. However, almost a half of the teenagers assessed in this study reported self-harm while being exposed to trigger sounds. It can be assumed that emotional distress and psychophysiological arousal related to misophonia may exceed the capabilities of younger children to respond in socially acceptable ways. Frequent verbal and physical aggression in children and teenagers with misophonia were also found in [6]. Our data indicate that externalizing behaviors in response to misophonic triggers might decrease with age, possibly along with an increase in self and social awareness and inhibitory control. However, as our data suggest, misophonia-related distress does not decrease and is frequently managed in a dysfunctional, self-destructive way, such as self-harm. Further, longitudinal studies should verify these results.

Despite the high rate of verbal and physical aggression reported in the younger misophonia sample, it is worth noting that there was no difference in the occurrence of ADHD and ODD between the misophonia group and the control group. The prevalence of ADHD in the misophonia group was very similar to that reported by Guzick et al. [6] when applying the DSM-5 criteria. In our study, 26% of the children met the criteria for some type of ADHD, while Guzick et al. [6] reported a prevalence of 21%. However, it is important to note that when applying the ICD-10 criteria, which are still official in Poland, the percentage of ADHD in the misophonia group dropped to 5%. Thus, it is crucial to consider the type of diagnostic criteria when comparing the data. Notably, [6] also found no significant difference in externalizing behavior between children with misophonia and those with anxiety disorders. What is more, Smit et al. [40] in the genome-wide association study did not find a genetic correlation between misophonia and aggression. However, they found significant correlations with the neuroticism cluster which holds internalizing traits. Therefore, our results, together with those from the other studies, support the notion that misophonia should be rather seen as an internalizing disorder. Externalizing behavior sometime observed in misophonia may be specifically related to the inability to overcome the psychophysiological response evoked by misophonic triggers, rather than a general way of reacting to various situations. This should also be verified in further studies, for example by comparing children with misophonia to those with externalizing disorders.

Clinicians should be aware that misophonia in children and adolescents can manifest in different ways and may not always involve externalizing behaviors such as verbal or physical aggression. Therefore, screening for self-harm and other related symptoms should also be a part of the diagnostic process for misophonia, particularly in cases where externalizing behaviors may not be apparent.

Another result of this study that indicates the need for careful psychiatric and psychological evaluation of children and teenagers with misophonia is related to depressive symptoms. Notably, when compared to controls without misophonia, children with misophonia on average had significantly higher self-reported depressive symptoms, but there was no difference in the parent reports. However, when analyzing the number of children whose results indicated clinically elevated depressive symptoms (according to norms adjusted for age in Poland), both self and parent reports indicated possible higher prevalence of this disorder in the misophonia group. A similar pattern of more apparent self-reported symptoms was found in the case of anxiety disorders. Only the symptoms of Generalized Anxiety Disorder were increased in the misophonia group according to both child and parent assessment, while symptoms of social phobia as well as panic and agoraphobia were significantly increased only when self-assessed by the children.

However, these results should be interpreted with caution for at least two reasons. Firstly, the majority of missing data in the depression and anxiety questionnaires was from younger children, many of whom were either too tired or unable to complete them. As a result, the mean age could be higher in the case of self-report compared to parental reports. Secondly, while we adjusted the data for age according to Polish norms for depressive symptoms, which makes the data more reliable in spite of the mentioned limitation, we used raw data for anxiety scales due to the lack of Polish norms for these questionnaires.

It is interesting to note the discrepancies in the assessment of OCD symptoms. Specifically, when children self-reported their OCD symptoms, there were no group differences. However, when assessed in the interview according to ICD-10 criteria, OCD was significantly more frequent in the misophonia group (13.6% vs. 0). In other words, the binary diagnosis made the difference statistically significant. Notably, in the dimensional parent-rated assessment of child OCD symptoms, although the difference was not statistically significant, the OCD symptoms were higher in children with misophonia, indicating a statistical tendency.In a study by [6], the diagnosis of OCD was also among the highest prevalent disorders (13%). These findings suggest that the use of multiple assessment methods as well as different sources of information, including self-report, parent report, and clinical interview may be useful for better identification of comorbid conditions in pediatric misophonia, especially in research, when the time for a diagnosis is more limited than in a clinical setting. The discrepancies between measurements, which also occurred in this study, can result from specific properties of the measurement tools, as well as from limited insight into their own psychopathology in children. It is also important for future studies to employ standardized interviews, such as MINI-KID [41], to ensure a higher quality of psychiatric assessment. In this study, due to constraints in resources, we were unable to do so.

The results showed that although children with misophonia had a relatively high rate of comorbid tic disorders (18%), there was no statistically significant difference compared to the control group (5%). However, due to time limitations, we did not use a dimensional scale to assess the severity of tic disorder symptoms, which could possibly show the whole spectrum and intensity of the phenomenon. Therefore, caution is advised when interpreting these results. Nonetheless, a similar (13%) rate of tic disorder in children with misophonia was discovered by Guzick et al. [6].

It was previously reported that misophonia might be related to migraines [7]. In our sample, children with misophonia indeed significantly more often experienced migraine or strong headaches, than controls, as reported by their parents. They also had a higher rate of psychosomatic complaints. The groups did not differ in occurrence of epilepsy, head injuries, and tinnitus. It should be further explored whether this correlation could be attributed to higher stress exposure or lower abilities of emotional regulation., such as for example emotional suppression.

In this study, we also aimed to investigate whether any prenatal, perinatal, or early childhood medical events could increase the risk of misophonia. We found no group differences in terms of delivery method, birth complications, prematurity, or maternal-reported stress during pregnancy with the assessed child. Nonetheless, these results also should be treated as preliminary and replicated with a use of more objective data, such as inclusion of medical records. Mothers of children with misophonia reported a significantly higher incidence of postpartum depression compared to mothers of children without misophonia. Further research is necessary to explore the emotional well-being of parents of children with misophonia, particularly regarding postpartum depression, as it has been already shown it may increase the risk of psychopathology [42, 43]. While we evaluated different aspects of parental functioning in this research project, it is not feasible to discuss them in detail within the scope of this paper due to its length.

Another finding that underscores the importance of focusing on families, both in terms of environmental and genetic factors, is that in over 50% of cases within the misophonia group, misophonia was found in at least one other member of the family. Although there are no twin studies on misophonia, some other data suggest a substantial impact of genetics factors. For example, the recent study by Smit et al. [40] showed 8.5% SNP-based heritability of misophonia. In this study, the prevalence of misophonia was not checked in the control group because participants were not chosen randomly from the population, and people who were already familiar with misophonia could volunteer for the study. To leverage knowledge on the family prevalence of misophonia, further studies should choose the comparison group randomly so that the epidemiological data are not biased.

Furthermore, in our misophonia group, the occurrence of autism in the family was high, although there were no significant group differences (30% vs. 16%). However, as mentioned above, there was a high risk that specific people might have applied to the control group, which could have biased the results. Therefore, further studies are needed to investigate the potential association between misophonia and autism in families using random sampling to reduce any potential bias. Particularly, misophonia symptoms could be explored in the context of the broader phenotype of autism [44]. Nonetheless, we found that children with misophonia did not differ significantly from the control group in terms of the severity of their ASD symptoms. In the largest study on children with misophonia to date, Guzick et al. [6] found that ASD symptoms were actually higher in the anxiety control group than in the misophonia group. Smit et al. [40] found even a negative correlation with autism in their study. In contrast, Rinaldi et al. [18] found a relationship between misophonia symptoms and ASD symptoms in both children and adults. However, the differences between their study and ours could be attributed to the broader definition of misophonia used by Rinaldi et al. [18] compared to the one defined by Jager et al. [3] as well as to the method of the data collections (questionnaire online vs. face-to-face interviews). The definition and measurement of misophonia can significantly impact its correlates, as demonstrated in Siepsiak et al. [8]. Therefore, when exploring the relationship between misophonia and ASD, it is crucial to carefully consider the definition and measurement of misophonia to ensure the accuracy of findings.

The outcome of our study, which found no relationship between misophonia and social difficulties measured by autism questionnaire assessment, is supported by the fact that the children with misophonia did not differ from their peers in tests measuring social competencies, nor were there differences in the test measuring emotional competencies. Further studies should examine whether the same results will be obtained in misophonia triggered primarily by non-oral or nasal human-made sounds, as this might be more related to sound over-responsivity prevalent in ASD, and using other measures of emotional and social functioning, including behavioral observations made by teachers.

A major limitation of this study is the small sample size. This problem could lead to the II type error, therefore—some differences between the groups could have not been detected. However, it should be noted that the study was intended to be a pilot study for a more comprehensive and expensive investigation into pediatric misophonia in Poland. Another significant limitation is the lack of a questionnaire to assess misophonia symptoms in children in Polish language, which prevented the exploration of the relationship between the severity of misophonia symptoms and other psychopathologies. Future research in Poland should prioritize the validation of existing child misophonia questionnaires (e.g. [17, 18, 45, 46]) to improve the accuracy of misophonia assessment in research and clinical practice. Another weakness of the study, which is also related to the lack of appropriate tools in Polish, is that we did not assess the comorbid hyperacusis in the misophonia group. We believe that this should be addressed in future studies.

There is also a scarcity of validated questionnaires in the Polish population for assessing other psychopathological symptoms in children. For instance, the SCAS was only validated in a group of teenagers and solely in the self-report version. Unfortunately, the cost of using better quality tools exceeded the budget of this pilot study. Furthermore, although there was no statistically significant age difference between the groups, it is worth noting that the misophonia group was, on average, one year older than the control group. In this developmental stage, even a one-year age difference can potentially result in significant qualitative differences, particularly when considering the prevalence of psychopathology symptoms. Regarding the age of onset of misophonia, this study provides only retrospective information; it would be valuable to conduct a longitudinal study where the younger siblings of children with misophonia are followed. Lastly, in future studies, the misophonia group should be compared to other clinical groups, such as a group of children with anxiety disorders [6] or externalizing disorders, to explore mechanisms that overlap with other psychopathologies and those specific to misophonia.

## Summary

The study showed that pediatric misophonia is related to increased psychopathology but not to ASD, ADHD or lower social and emotional skills. It also provided additional rationale for characterizing misophonia more as an internalizing rather than externalizing disorder. The data highlights the importance of further investigation into the developmental factors of misophonia and underscores the need for more comparative studies where misophonia correlates are compared to for example ASD, hyperacousis or externalizing disorders. Additionally, longitudinal studies and those involving family characteristics would be beneficial in gaining a more profound understanding of misophonia.

## Supplementary Information

Below is the link to the electronic supplementary material.

Supplementary file1 (PDF 113 kb)

Supplementary file2 (PDF 198 kb)

## Data Availability

The data will be made available for any research purposes upon request (at marta.siepsiak@psych.uw.edu.pl).
