# Supplementary material for: Misophonia in Children and Adolescents: Age Differences, Risk Factors, Psychiatric and Psychological Correlates. A Pilot Study with Mothers’ Involvement
Source: Child Psychiatry Hum Dev. 2023 Sep 8;56(3):758–71. doi: 10.1007/s10578-023-01593-y (PMC12095346; doi:10.1007/s10578-023-01593-y)
Supplement: Supplementary file 1 — Supplementary file1 (PDF 113 kb) [file 10578_2023_1593_MOESM1_ESM.pdf]

Appendix 1.

Code for the participant [ ] gender [ ]
place of living, voivodeship………………………………….. age [ ]

I. Misophonia interview (on the basis of Jager i in., 2020 criteria).

1. Are there any sounds that evoke difficult emotions in your child?

   No → go to the Health Interview (II)

   Yes → What kind of sounds?

[ ] eating/smacking sounds [ ] breathing sounds [ ] sniffling sounds

[ ] normal chatting [ ] sounds behind the wall [ ] various loud sounds

[ ] sudden sounds

[ ] other : **……………………………………………………………………………………………………**

2. How can you tell if a certain sound causes suffering to a child? (asking for examples of specific situations)

   ……………………………………………………………………………………………………
   ……………………………………………………………………………………………………

3. Does the child talk about what they are experiencing? [Note: We did not report this anywhere. The question was included here just in case, but in fact, the major information was already provided in the previous question.)

   ……………………………………………………………………………………………………
   ……………………………………………………………………………………………………

4. What does the child feel when they hear these sounds? / What do you feel when you hear these sounds?

   I don't know [ ]

   fear [ ] anger [ ] panic [ ] disgust[ ] anxiety [ ] concern [ ]

   irritation [ ] frustration [ ] other:
   ………………………………………………………………………………………

5. How quickly does the reaction to the sound appear:

even before it is emitted, when the child sees that the sound is about to occur [ ]

immediately, within 1 to 5 seconds [ ]

within about a minute [ ]

after about 5 minutes of continuous the repetitive sound [ ]

other: [ ]……………………………………………………………………………………..

6. What does the child/do you most often do when they/you hear these noises at home/family environment?

calmly asks to stop [ ]   leaves the room [ ]

screams to stop [ ]   nervously asks to stop [ ]

hits someone [ ]   hits something [ ]

hurts themselves (e.g., pinching, hitting themselves) [ ]

covers their ears with their hands [ ] puts on headphones [ ]

Does the child do something else? ……………………………………………………………

7. How old was the child when the sounds started bothering them?……………………………………………………………………………………………………
8. What was the first sound that caused such a reaction? What/Who made it?…………………………………………………………………………………………………...
9. Since that time, the reactions:

intensify [ ]   remain the same [ ]   diminish [ ]   hard to say [ ]

10. Since that time, the number of difficult sounds:

increases [ ]   remains the same [ ]   decreases [ ]   hard to say [ ]

11. Reactions to sounds made by family members are:

stronger than to sounds made by people outside the family [ ]

the same for family and non-family members [ ]

other response: [ ]……………………………………………………………………….

12. Does anyone in the family react in a similar way to sounds? / Do they react strongly to certain rather quiet, repetitive sounds made by a person? [Note: only sibling, parents, grandparents and uncles/aunts were reported - siblings of parents who shared at least one grandparent with the assessed child]

    Who? ……………………………………………………………………………………………..

13. On a scale from 0 to 10, please indicate how much the child's sound sensitivity disrupts family functioning, where 0 means not at all, and 10 means completely disrupting family life.

    parent: 1 2 3 4 5 6 7 8 9 10

To what extent does sound sensitivity affect your life? This includes how you feel, concentrate, your relationships with friends and classmates, etc. [this item was not analyzed in this paper]

child: 1 2 3 4 5 6 7 8 9 10

Comments:..

II. Health Interview

1. **Labour**

Natural [ ] CC [ ]

On time [ ] late [ ] premature [ ] complications [ ] Apgar [ ]

Comments: …………………………………………………………………………………………

2. **Postpartum depression [ ]**

It is normal for a woman to feel significantly worse for a few days after giving birth, and sometimes a low mood persists for up to 2 weeks, known as "baby blues." However, sometimes postpartum depression can occur. Based on what you remember, do you think you could have had postpartum depression, which is a state of significantly lowered mood that appeared around the first 6 weeks after giving birth and lasted longer than 2 weeks? Please describe what was happening.

3. **Stress during pregnancy [ ]**

In your opinion, was the period of pregnancy with xxx associated with particular stress? Did any stressful events or situations happen?

4. **Autism in family yes [ ] no [ ]**

According to your knowledge, does anyone in the family have a diagnosis of autism spectrum disorder, including Asperger Syndrome? [Note: only sibling, parents, grandparents and uncles/aunts

were reported - siblings of parents who shared at least one grandparent with the assessed child]

5. **Do your child have:**

Migraines [ ]

Severe headaches, unrelated to any diagnosed medical condition, lasting even up to several hours, may also be accompanied by nausea or sensitivity to light.

Head injuries [ ]

Occurred before the sound over responsivity [for misophonia group], *with incidents of loss of consciousness, dizziness, blurred vision, vomiting, or nausea.*

Epilepsy (officially diagnosed) [ ]

tinnitus [ ]

dyslexia (officially diagnosed) [ ]

psychosomatic complaints [ ]

Does your child complain, such as unexplained, without documented medical reasons, stomach pain, back pain, or pain in other parts of the body? Please describe it. [Note: the physiological component of emotions, such as stomach pain right before or during an exam, was not counted as psychosomatic complaints]

6. **Other health issues:**

....
....
....
....
....

Interview - Obsessive-Compulsive Disorder Checklist CY-BOCS

"COMPULSIONS are all the actions that you feel compelled to perform, even though you know that such behavior makes no sense. Usually, giving into compulsions aims to reduce anxiety related to the mental suffering caused by obsessive thoughts."

Here is a list of the most common compulsions (symptom list from the CY-BOCS scale). Does any of them occur in the child? NO [ ] YES → which one?

....

Did the symptom persist for more than 2 weeks? YES NO

Did the symptom persist for more than 12 months? YES NO

"OBSESSIONS are intrusive, recurrent, and distressing thoughts, sensations, urges, or images. They can be realistic or unrealistic and usually evoke anxiety."

Here is a list of the most common obsessions (symptom list from the CY-BOCS scale). Does any of them occur in the child? NO [ ] YES → which one?

....

Did the symptom persist for more than 2 weeks? YES NO

Did the symptom persist for more than 12 months? YES NO

TIC Disorder INTERVIEW

Does the child have tics? Tics are repetitive, involuntary movements or vocalizations. Sometimes they may appear to be purposeful.

Here is a list of the most common tics and compulsive behaviors associated with tics (tic list based on the Yale Global Tic Severity Scale YGTSS).

Did the symptoms occur in the child for at least 4 weeks? [ ]

Did they persist for over 12 months? [ ]

III. Other

Mean of grades at school ………….

Grade for behavior at school ……………

→ ADHD Interview - Appendix 2 (Polish version, the interview is entirely and directly based on ICD-10 criteria).
