# Supplementary material for: Misophonia in Children and Adolescents: Age Differences, Risk Factors, Psychiatric and Psychological Correlates. A Pilot Study with Mothers’ Involvement
Source: Child Psychiatry Hum Dev. 2023 Sep 8;56(3):758–71. doi: 10.1007/s10578-023-01593-y (PMC12095346; doi:10.1007/s10578-023-01593-y)
Supplement: Supplementary file 2 — Supplementary file2 (PDF 198 kb) [file 10578_2023_1593_MOESM2_ESM.pdf]

Kwestionariusz ustrukturyzowanego wywiadu diagnostycznego w kierunku zespołu nadpobudliwości psychoruchowej (ADHD) według ICD-10 i DSM-IV TR

Imię i nazwisko dziecka:			
Data badania:		*Ocenia okres:**	
Przyjmowane leki:			

** PRZY PIERWSZORAZOWYM BADANIU OCENIAMY WYMAGANY DO ROZPOZNANIA OKRES 6 M-CY, PRZY KOLEJNYCH W ZALEŻNOŚCI OD POTRZEBY – – „2 M-CE OD OSTATNIEJ WIZYTY", „1 M-C OD WPROWADZENIA LEKU". MINIMUM CZASU OCENIANEGO TO 2 TYGODNIE.*

ZABURZENIA UWAGI

1. Dziecko często nie jest w stanie utrzymać ciągłej i bliższej uwagi na szczegółach lub popełnia błędy z nieuwagi w szkole, pracy lub innej działalności.
   tak (3) raczej tak (2) chyba nie (1) nie (0)
2. Dziecku często nie udaje się utrzymać trwałej uwagi na zadaniach lub grach/zabawach.
   tak (3) raczej tak (2) chyba nie (1) nie (0)
3. Często wydaje się, że dziecko nie słyszy tego, co się do niego/niej mówi.
   tak (3) raczej tak (2) chyba nie (1) nie (0)
4. Dziecko często nie jest w stanie wykonać następujących po sobie instrukcji lub skończyć pracy szkolnej, zadanej pracy lub obowiązków w miejscu pracy (ale nie z powodu przeciwstawiania się (opozycyjnego zachowania) lub niezrozumienia instrukcji).
   tak (3) raczej tak (2) chyba nie (1) nie (0)
5. Dziecko często ma trudności ze zorganizowaniem sobie pracy lub innych zajęć.
   tak (3) raczej tak (2) chyba nie (1) nie (0)
6. Dziecko nie lubi, ociąga się lub unika zajęć wymagających dłuższego wysiłku umysłowego – jak nauka szkolna lub odrabianie zajęć domowych.
   tak (3) raczej tak (2) chyba nie (1) nie (0)
7. Dziecko często gubi rzeczy niezbędne do pracy lub innych zajęć, np.: zabawki, przybory szkolne, ołówki, książki, narzędzia.
   tak (3) raczej tak (2) chyba nie (1) nie (0)
8. Dziecko łatwo rozprasza się pod wpływem zewnętrznych bodźców.
   tak (3) raczej tak (2) chyba nie (1) nie (0)
9. Dziecko często jest zapominalskie w trakcie codziennych zajęć.
   tak (3) raczej tak (2) chyba nie (1) nie (0)

Liczba objawów		*Liczba punktów objawowych*	

NADRUCHLIWOŚĆ

1. Dziecko ma często nerwowe ruchy rąk lub stóp bądź nie jest w stanie usiedzieć w miejscu, często macha rękami lub nogami albo wierci się na krześle.
   tak (3) raczej tak (2) chyba nie (1) nie (0)
2. Dziecko wstaje z miejsca w czasie lekcji lub w innych sytuacjach wymagających spokojnego siedzenia.
   tak (3) raczej tak (2) chyba nie (1) nie (0)
3. Dziecko często biega wkoło lub wchodzi na meble w sytuacjach, w których jest to nieaprobowane (u nastolatków lub dorosłych może być obecne tylko uczucie niepokoju).
   tak (3) raczej tak (2) chyba nie (1) nie (0)
4. Dziecko często jest nadmiernie hałaśliwe w zabawie i ma trudności ze spokojnym bawieniem się.
   tak (3) raczej tak (2) chyba nie (1) nie (0)
5. Dziecko często jest w ruchu; „biega jak nakręcone". Zachowanie dziecka cechuje nadmierna aktywność ruchowa, na którą nie wpływa ani sytuacja, ani potrzeba.
   tak (3) raczej tak (2) chyba nie (1) nie (0)

Liczba objawów		*Liczba punktów objawowych*	

IMPULSYWNOŚĆ

6. Dziecko często wyrywa się z odpowiedzią, zanim pytanie zostanie zadane do końca.
   tak (3) raczej tak (2) chyba nie (1) nie (0)
7. Dziecku często nie udaje się stać w szeregu lub poczekać na swoją kolej w grze lub sytuacji grupowej.
   tak (3) raczej tak (2) chyba nie (1) nie (0)
8. Dziecko często przerywa lub przeszkadza innym (np. wtrąca się do rozmowy lub zabawy innych osób).
   tak (3) raczej tak (2) chyba nie (1) nie (0)
9. Dziecko często jest nadmiernie gadatliwe.
   tak (3) raczej tak (2) chyba nie (1) nie (0)

Liczba objawów		*Liczba punktów objawowych*	

B. Niektóre upośledzające funkcjonowanie dziecka objawy zaburzeń koncentracji uwagi lub nadpobudliwości psychoruchowej (nadruchliwości, impulsywności) ujawniły się przed 7. rokiem życia dziecka.
   tak (3) raczej tak (2) chyba nie (1) nie (0)

C. Upośledzenie funkcjonowania dziecka spowodowane tymi objawami występuje w dwóch lub więcej sytuacjach (np. w szkole i w domu).
   tak (3) raczej tak (2) chyba nie (1) nie (0)

D. Stwierdza się klinicznie istotne upośledzenie funkcjonowania społecznego, zawodowego lub szkolnego (w zakresie edukacji).
   tak (3) raczej tak (2) chyba nie (1) nie (0)

E. Objawy u dziecka nie występują w przebiegu przetrwałych zaburzeń rozwojowych, schizofrenii lub innych psychoz i nie można ich trafniej uznać za objawy innego zaburzenia psychicznego (np. zaburzeń nastroju, lękowych, dysocjacyjnych lub nieprawidłowej osobowości).
   tak (3) raczej tak (2) chyba nie (1) nie (0)

**Rozpoznanie według ICD-10:**	
**Rozpoznanie według DSM- IV:**	

© Wolańczyk, Kołakowski, 2005-2011
Kwestionariusze mogą być używane jedynie do celów klinicznych. Kwestionariusz do wypełnienia podczas rozmowy diagnostycznej.

Kwestionariusz ustrukturyzowanego wywiadu diagnostycznego w kierunku zaburzenia opozycyjno-buntowniczego (ODD) i zaburzeń zachowania (CD) według DSM-IV-TR

Imię i nazwisko dziecka:			
Data badania:		*Ocenia okres 6 miesięcy:*	*Ocenia okres 12 miesięcy:*
Przyjmowane leki:			

ODD

1. Miewa częste lub nadmiernie nasilone wybuchy złości w stosunku do swojego poziomu rozwoju.
   tak (3) **raczej tak (2)** **chyba nie (1)** **nie (0)**
2. Często spiera się/kłóci z dorosłymi.
   tak (3) **raczej tak (2)** **chyba nie (1)** **nie (0)**
3. Często czynnie sprzeciwia się dorosłym lub lekceważy normy.
   tak (3) **raczej tak (2)** **chyba nie (1)** **nie (0)**
4. Często w sposób widoczny umyślnie robi rzeczy, które sprawiają przykrość innym ludziom.
   tak (3) **raczej tak (2)** **chyba nie (1)** **nie (0)**
5. Często obwinia innych za swoje błędy lub złe zachowanie.
   tak (3) **raczej tak (2)** **chyba nie (1)** **nie (0)**
6. Często jest przewrażliwione lub łatwo je urazić.
   tak (3) **raczej tak (2)** **chyba nie (1)** **nie (0)**
7. Często się złości lub obraża.
   tak (3) **raczej tak (2)** **chyba nie (1)** **nie (0)**
8. Bywa często złośliwe lub mściwe.
   tak (3) **raczej tak (2)** **chyba nie (1)** **nie (0)**

Liczba objawów		*Liczba punktów objawowych*	

CD

a) Często znęca się nad innymi (np. umyślnie zadaje ból, włącza się w to także przewlekłe zastraszanie, zadawanie cierpienia lub molestowanie).
   tak (3) **raczej tak (2)** **chyba nie (1)** **nie (0)**
b) Często rozpoczyna bójki (nie licząc bójek z rodzeństwem).
   tak (3) **raczej tak (2)** **chyba nie (1)** **nie (0)**
c) Choć raz użył broni, która mogła wyrządzić komuś poważna krzywdę (np. kija, cegły, stłuczonej butelki, noża, broni palnej).
   tak (3) **raczej tak (2)** **chyba nie (1)** **nie (0)**
d) Choć raz okazał fizyczne okrucieństwo wobec innych osób (np. związał, pokaleczył lub podpalił ofiarę).
   tak (3) **raczej tak (2)** **chyba nie (1)** **nie (0)**
e) Był okrutny fizycznie wobec zwierząt.
   tak (3) **raczej tak (2)** **chyba nie (1)** **nie (0)**
f) Choć raz popełnił przestępstwo wymagające konfrontacji z ofiarą (wyrwanie portfela, wymuszenie, rozbój).
   tak (3) **raczej tak (2)** **chyba nie (1)** **nie (0)**
g) Choć raz zmusił inną osobę do aktywności seksualnej.
   tak (3) **raczej tak (2)** **chyba nie (1)** **nie (0)**
h) Choć raz umyślnie wzniecił pożar z ryzykiem lub zamiarem spowodowania poważnego zniszczenia.
   tak (3) **raczej tak (2)** **chyba nie (1)** **nie (0)**
i) Choć raz umyślnie zniszczył własność innych (inaczej niż przez podpalenie).
   tak (3) **raczej tak (2)** **chyba nie (1)** **nie (0)**
j) Choć raz włamał się do czyjegoś mieszkania, domu lub samochodu.
   tak (3) **raczej tak (2)** **chyba nie (1)** **nie (0)**
k) Często kłamie lub łamie obietnice, aby uzyskać korzyść lub uniknąć zobowiązań.
   tak (3) **raczej tak (2)** **chyba nie (1)** **nie (0)**
l) Kradnie przedmioty o znacznej wartości bez konfrontacji z ofiarą, zarówno w domu, jak poza nim (np. kradzieże w sklepach, włamania, fałszerstwa).
   tak (3) **raczej tak (2)** **chyba nie (1)** **nie (0)**
m) Często pozostaje w nocy poza domem, mimo zakazu rodziców. (Rozpoczął przed ukończeniem 13 roku życia).
   tak (3) **raczej tak (2)** **chyba nie (1)** **nie (0)**
n) Uciekł z domu lub z domu rodziny zastępczej przynajmniej dwukrotnie bądź raz dłużej niż na jedną noc (z wyjątkiem ucieczki w celu uniknięcia fizycznego lub seksualnego maltretowania).
   tak (3) **raczej tak (2)** **chyba nie (1)** **nie (0)**
o) Często opuszcza lekcje w szkole (wagaruje). (Rozpoczął przed ukończeniem 13 roku życia).
   tak (3) **raczej tak (2)** **chyba nie (1)** **nie (0)**

Liczba objawów		*Liczba punktów objawowych*	

A. Objawy powodują klinicznie znaczące upośledzenie w społecznym, edukacyjnym lub zawodowym funkcjonowaniu pacjenta.
   Tak **Nie**
B. Czy pacjent powyżej osiemnastego roku życia spełnia kryteria osobowości antyspołecznej?
   Tak **Nie**
C. Czy zaburzenie spełnia kryteria dla osobowości antyspołecznej, schizofrenii, epizodów manii lub depresji, przetrwałych zaburzeń rozwojowych, zespołu hiperkinetycznego?
   Tak **Nie**
D. Czy spełnione są kryteria zaburzeń emocjonalnych (lek, depresja, natręctwa, hipochondria)?
   Tak **Nie**
E. Czy pacjent spełniał przynajmniej jedno kryterium przed ukończeniem 10 roku życia?
   Tak = typ z początkiem w dzieciństwie **Nie = typ z początkiem w okresie dorastania**

**Brak rozpoznania**	**ODD**	**CD (klinicznie + ODD)**	**CD**
